# Italian Technostress Creators Scale: Validation of an Assessment Tool

**DOI:** 10.1097/JOM.0000000000003478

**Published:** 2025-06-05

**Authors:** Maria Francesca Rossi, Flavia Beccia, Francesco Iantorno, Giuseppe Melcore, Maria Rosaria Gualano, Umberto Moscato

**Affiliations:** From the Department of Health Science and Public Health, Catholic University of the Sacred Heart, Rome, Italy (M.F.R., F.B., F.I., G.M., U.M.); Saint Camillus International University of Health Sciences, UniCamillus, Rome, Italy (M.R.G.); and Department of Woman and Child Health and Public Health, Public Health Section, Fondazione Policlinico Universitario A. Gemelli IRCCS, Rome, Italy (U.M.).

**Keywords:** Technostress Creators Scale, technostress, validation, occupational health

## Abstract

**Objective:**

The aim of this study was to develop and validate a tool to assess technostress in Italian workers.

**Methods:**

A questionnaire was developed by back-translating the Technostress Creators Scale, adding sociodemographic questions, investigating work characteristics, frequency of technology use, etc. Exploratory factor analysis examined the underlying factor structure. Model fit was evaluated through confirmatory factor analysis.

**Results:**

The sample consisted of 90 participants, 53.3% female, mean age 37.4 (standard deviation ±10.9) years. Exploratory factor analysis revealed a six-factor structure explaining 58% of the variance, with factor loadings >0.40. CFE showed good fit indices (*χ*^2^/*degrees of freedom* = 4.02, comparative fit index = 0.926, Tucker-Lewis index = 0.915, root mean square error of approximation = 0.057, standardized root mean square residual = 0.079).

**Conclusions:**

The Italian version of the Technostress Creators Scale showed good reliability. This validation is a starting point to develop new strategies to assess, evaluate, and prevent technostress in the workplace.

## Abstract 2

This study validated a tool to assess technostress in Italian workers. Due to technostress’ rising impact, this could be fundamental for occupational physicians to assess work-related technostress during clinical practice. Recognizing and assessing this issue will be crucial to improve the occupational well-being and mental health of workers.

## Graphical abstract

**Figure:**
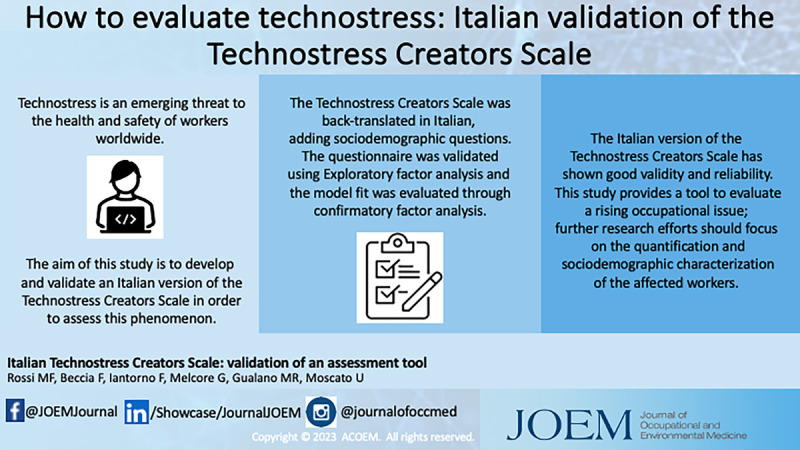

**LEARNING OUTCOMES**

- The Italian validation of the Technostress Creators Scale will allow occupational physicians to perform an assessment of work-related technostress in Italian workers, thus ensuring their the psychological well-being.
- The factorial analyses confirmed the appropriateness of the original scale, at the same time underlining a convergence of the first two factors, which could lead to the development of a shorted (and easier to administer) version of this questionnaire.
- This study provides a much needed tool to evaluate a rising occupational issue and provides an insight into the technostress phenomenon.
- Further research efforts should focus on a quantification of the issue and on investigating the sociodemographic characteristics of the affected workers.

During the COVID-19 pandemic, starting in 2020, many workers were forced to change their lifestyles, as many of them started to work from home (WFH) to prevent the spread of SARS-CoV-2. WFH consists of having a remote work location and the use of information and communication technologies (ICTs) to work. This rapid shift of working environment left many people with little information or guidance on how their jobs would change. This situation created both opportunities and additional sources of potential stress and conflict for employees.^1–3^

Many studies investigated the effects of WFH on workers’ health, concerning both the physical and mental well-being. Xiao e al. in 2021 reported that approximately two-thirds (64.8%) of respondents had new physical health issues, and approximately three-fourths (73.6%) had new mental health issues arising since WFH.^4^

Regarding the physical health, there has been an increase in musculoskeletal pathologies. Computer use is associated with extended static postures, repetitive movements, and wrist and forearm fatigue. These factors are associated with the development of musculoskeletal symptoms and disorders within the neck, wrist, shoulders, hands, and lower back.^5^

Furthermore, the COVID-19 pandemic itself has had a negative impact on the general and mental health of workers worldwide^6,7^ and in Italy.^8^ The effect of the pandemic on workers’ mental health was even more prominent for remote workers with inflexible schedules, precarious workers, or those who lost their jobs.^9^ In Italy, COVID-19 affected many workers in a variety of sectors,^10^ with important health consequences^8^; many workers had difficulties resuming their work activities after suffering from COVID-19.^11,12^ Overall, the COVID-19 pandemic has highlighted the need to implement public health interventions in the workplace to foster the physical and mental well-being of workers.^13^

On the other hand, recent scientific researches have highlighted the negative consequences on mental health of intense ICT use at work, including lower job satisfaction and productivity, increased burnout, and work exhaustion.^14^ In a systematic review conducted by Gualano et al.,^15^ 78.9% of included studies reported high levels of telework-related stress in remote workers. Furthermore, the authors underline the emerging of new psychosocial risks due to the improvement and diffusion of ICTs.

Technostress is the term used to indicate the inability to interact with ICT in a healthy manner.^16^ In 1997, Weil and Rosen^17^ modified the definition of technostress considering it as “any negative impact on attitudes, thoughts, behaviors, or body physiology that is caused either directly or indirectly by technology.” Finally, Ragu-Nathan et al. in 2008 assume technostress “is caused by an individual’s attempts to deal with constantly evolving ICTs and the changing physical, social, and cognitive responses demanded by their use.” The importance of technostress in negatively affecting worker’s health and its social impact has been correlated to the dependence on ICTs and because of the increasing sophistication of ICTs,^18^ leading workers to constantly update their technological knowledge.

The Technostress Creators Scale (TCS) is a measuring instrument used to assess technostress. It was developed and validated by Ragu-Nathan et al. in 2008. The tool consists of a 5-point Likert-type scale, which measures five items: techno-overload, technoinvasion, technocomplexity, technoinsecurity, and technouncertainty. These five items represent the Technostress Creators described for the first time by Tarafdar et al. in 2007.^19^ Technostress Creators are the factors positively associated with technostress in the organization, such as the constant connectivity, the communication and information overload, and the competitive pressures to keep using the latest hardware, software, and applications.^18^

Technostress is increasingly affecting workers worldwide and therefore posing a threat to the health and safety of Italian workers. The Italian Legislative Decree 81/08, which regulates health and safety in the workplaces, states that the risk assessment must consider everything that constitutes a risk for the safety and health of workers, including work-related stress.^20^ In Italy, technostress has been investigated mainly through ad hoc questionnaires, highlighting a strong impact of this condition on the health of workers during the COVID-19 pandemic, especially in those who worked remotely.^21,22^ Furthermore, technostress has been reported to affect female workers more than males^23–25^ and to be higher in older workers.^15,25^ According to a recent national survey, 28% of the Italian working population suffers from technostress.^26^ Therefore, it is clear that an Italian instrument to measure technostress in Italy is needed.

The main aim of this study was to develop and validate an Italian version of the TCS questionnaire. Furthermore, this study aims to develop a shortened version of the scale, adapted to the working Italian population, to provide Italian occupational health physicians with a tool they can use during clinical practice to assess the workers’ risk of technostress.

## METHODS

The original TCS created and validated by Ragu-Nathan et al.^18^ was back-translated from English to Italian. One of the authors translated the original version, another author back-translated the Italian version, and a third author compared the two English versions to ensure that consistency and meaning were maintained during translation. The resulting questionnaire in Italian was then adapted to fit the target population, removing the three original items investigating end-users knowledge. The three items were removed to adapt the questionnaire to the Italian working population and to work sectors who do not have end-users, such as the healthcare sector, so that the validation of the questionnaire could be performed in the entirety of the Italian working population to ensure the adaptability for future uses.

Furthermore, six items were added to investigate work location, frequency of technology use at work, training pertaining to technology, and technology perception, to investigate work context and relate it to the TCS, to assess the influence of work contexts on technostress.

### Sample

Participants were recruited to participate in the study if they fit the inclusion criteria: being older than 18 years, working in Italy at the time of the survey, using ICTs during their routine work tasks, and giving their informed consent to participate in the study.

Recruitment was done through snowball methodology selecting a sample from diverse background and demographics, to ensure that the sample was representative of the Italian working population. Participants from different Italian Regions were recruited, distributing the questionnaire to people from North, Center, and South Italy.

The study was approved by the local ethics committee (Comitato Etico Lazio Area 3, ID protocol no. 6339).

### Questionnaire

The resulting questionnaire in Italian language is structured into four sections: the first section is aimed at investigating the sociodemographic characteristics of the participants (eight items) and gathers information concerning gender, age, and geographical area of the participants. Concerning the respondent’s profession, the Italian National Statistics Institute classification was used in the option’s list to ensure a nationally recognized classification.

The second section investigates technostressors (20 items), and the third section investigates technoinhibitors (four items). Sections 2 and 3 of the questionnaire were developed by back-translating the TCS questionnaire by Ragu-Nathan et al.^18^

The fourth section aimed at investigating the worker’s adaptation to the use of technology (six items). In this section, questions assessing the participants’ work location, frequency of technology use at work, and training pertaining to technology were developed by experts in the field of technostress and work-related stress and were then added to the questionnaire.

### Statistical Analysis

To assess the internal reliability of the instrument, interitem correlations were assessed, and a leave-one-out analysis was performed to determine whether the reliability coefficients improved with the deletion of any individual item.^27^ Interitem correlations and corrected item-total correlations between 0.3 and 0.7 were considered satisfactory.^28,29^ Cronbach *α* (C*α*) coefficients were calculated, and coefficient of 0.70 or higher was deemed indicative of high reliability.^30^ Given the ordinal nature of the scale items, McDonald’s *ω* reliability coefficients were also calculated.^31^

After confirming the factorability of the correlation matrix, an exploratory factor analysis (EFA) was conducted. The adequacy of the factor solution was evaluated using Bartlett test of sphericity.^32^ To investigate construct validity, latent factors were extracted through EFA, following the recommendations of Ledesma et al.^33^ Principal axis analysis with oblique rotation (promax) was used to allow for correlation among the extracted factors, assuming factor interdependence.^34^ The Kaiser-Meyer-Olkin (KMO) measure was calculated, with KMO values >0.60 considered suitable for factor analysis.^35^ KMO values ranging from 0.70 to 0.80, and 0.80 to 0.90 were interpreted as good and excellent, respectively.^36,37^ To determine the appropriate number of factors, we used both Kaiser’s criterion and a scree plot.^32^

Latent factors were extracted using maximum likelihood estimation with promax rotation. A minimum factor loading of 0.3 was considered significant.^38^ Items with pattern coefficients of 0.40 or greater were retained, whereas those with coefficients less than 0.40 across all factors or cross-loading on two or more factors with values of 0.32 or greater were removed. Each time an item was removed, the EFA process was repeated.^39^

Confirmatory factor analysis (CFA) was then used to verify the alignment between the scale’s factor structure and the hypothesized model from the EFA. The final model was improved by checking modifications indices. Covariances were added if the modification was greater than 20, and variances were within the same construct.^40^

The maximum likelihood estimation method was used for the CFA. The model’s fit was assessed using multiple indices, including the adjusted *χ*^2^/*degrees of freedom* (*df*), root mean square error of approximation (RMSEA), root mean square residual, comparative fit index, and Tucker-Lewis index.^41^ The thresholds for model fit acceptance are provided in Table 1,^37^ and the *χ*^2^/*df* value less than 5 was considered acceptable.^42^ Adopting Hu and Bentler’s Two-Index Presentation Strategy, RMSEA and standardized root mean square residual were used to describe the final model.^42^

**TABLE 1:** Thresholds for Model Fit Acceptance

Fit Indices	Acceptable Rate
Root mean square error of approximation	<0.08
Standardized root mean square residual	<0.08
Comparative fit index	>0.9
Tucker-Lewis Index (nonnormed fit index)	>0.9
Minimum discrepancy function by degrees of freedom divided	<5
*χ*^2^ *P* value	>0.05

Statistical analyses were performed using STATA 18 software.^43^

## RESULTS

The final sample consisted of 90 participants, 48 were female (53.3%), and the mean age was 37.4 (standard deviation ±10.9). Main characteristics of the sample are summarized in Table 2.

**TABLE 2:** Sociodemographic Variables of Included Participants

	No. of Participants	Mean (±SD)
Age, y	90	37.4 (±10.9)
	**No. of participants**	**Percentage**
Gender		
Male	42	46.7
Female	48	53.3
Marital status		
Unmarried	42	46.7
Married/cohabiting	48	53.3
Children		
None	62	68.9
One	12	13.3
More than one	16	17.8
Education		
High school diploma	12	13.3
College degree	64	71.1
Post-gradual degree	14	15.6
Job		
Highly specialized profession	58	64.4
Technical profession	4	4.4
Service industry	11	12.2
Office workers	13	14.4
Managers, entrepreneurs, legislators	2	2.2
Other	2	2.2
Work location		
On site only	32	35.6
On site most of the time	38	42.2
Half remotely and half on site	13	14.4
Remotely most of the time	7	7.8
Technology use at work		
Every day	86	95.6
At least 3 d per week	2	2.2
At least 1 d per week	2	2.2
Technology training course		
Never participated	31	34.4
Participated privately	21	23.3
Participated through company	38	42.2

Concerning education and training variables, most participants’ higher education had college degree (71.1%), and most participants were married (53.3%) and did not have children (68.9%).

The majority of the participants worked on site most of the time (42.2%) and used technology while performing work tasks every day (95.6%). Most workers had participated in a training course concerning technology (42.2%) through their company.

The analysis suggested satisfactory reliability (internal consistency) of the Italian version of the TCS, with C*α* and *ω* values of 0.881 and 0.887, respectively. No consistent increases in reliability were observed in the leave-one-out analyses. Mean, standard deviation, and C*α* and *ω* for the entire scale are presented in Table 3. At the item level, interitem correlations and question exclusion analysis (leave-one-out) are also reported.

**TABLE 3:** Descriptive Statistics, Validity, and Reliability of the Italian Version of the Technostress Creators Scale (TCS) and Each Item of the Scale

	Mean	Standard Deviation	Interitem Correlation	Cronbach *α*	McDonald’s *ω*
TCS	3.59	0.517	—	0.881	0.887
Q1	3.44	1.237	0.584	0.873	0.880
Q2	3.51	1.192	0.567	0.874	0.880
Q3	3.48	1.124	0.607	0.873	0.879
Q4	3.61	1.148	0.647	0.871	0.878
Q5	3.48	1.238	0.337	0.880	0.885
Q6	4.03	1.065	0.536	0.875	0.881
Q7	2.91	1.269	0.452	0.877	0.883
Q8	3.72	0.912	0.592	0.874	0.879
Q9	3.88	0.872	0.610	0.874	0.879
Q10	3.32	1.037	0.482	0.876	0.882
Q11	3.57	0.995	0.452	0.877	0.883
Q12	3.87	0.864	0.560	0.875	0.880
Q13	4.07	0.981	0.461	0.877	0.883
Q14	3.91	0.932	0.577	0.874	0.879
Q15	4.03	1.075	0.575	0.874	0.880
Q16	4.26	0.966	0.459	0.877	0.882
Q17	3.00	0.994	0.423	0.877	0.884
Q18	3.26	0.931	0.480	0.876	0.882
Q19	3.57	0.972	0.474	0.876	0.882
Q20	3.57	0.984	0.439	0.877	0.883
Q21	3.33	1.071	0.243	0.882	0.888
Q22	2.62	1.118	0.180	0.884	0.889
Q23	3.51	1.104	0.386	0.878	0.884
Q24	3.20	1.163	0.335	0.880	0.886
Q25^a^	3.81	0.982	0.138	0.884	0.889
Q26^a^	4.28	0.862	0.132	0.883	0.889
Q27^a^	3.69	0.956	0.101	0.884	0.890

Cronbach *α* and McDonald’s *ω* for each item are calculated as if the item if left out (leave-one-out analysis).

^a^Item is on an inverted scale; these three items are questions regarding interest in technology added to the back-translated scale.

**FIGURE 1:**
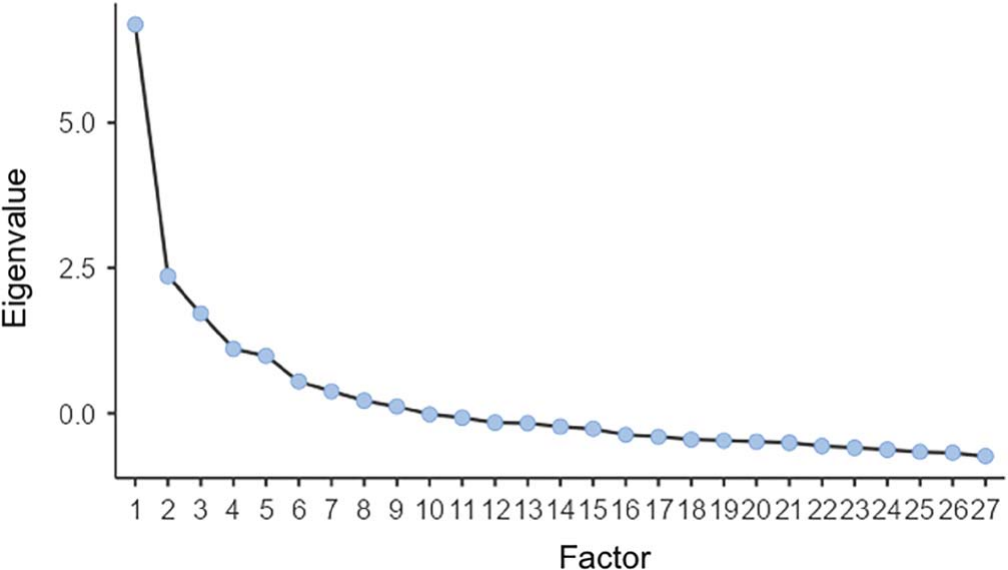
Exploratory factor analysis: scree plot.

The KMO value was 0.78, indicating adequacy of the sample for further analysis. The result of Bartlett test of sphericity was also significant (*P* < 0.001, *χ*^2^ = 1353, *df* = 351). EFA extracted a six-factor model. Item loadings and uniqueness are reported in Table 4. Both the scree plot (Fig. 1) and the cumulative percentage of variance (Table 5) indicated the model could be considered suitable.

**TABLE 4:** Exploratory Factor Analysis: Item Loadings and Uniqueness

	Factors						Uniqueness
	1	2	3	4	5	6		
Q1	0.787						0.394
Q2	0.759						0.438
Q3	0.740						0.345
Q4	0.636						0.359
Q5	0.633						0.549
Q6	0.609						0.479
Q7	0.407						0.728
Q8		0.979					0.141
Q9		0.951					0.186
Q10		0.815					0.304
Q11		0.407					0.564
Q12			0.918				0.149
Q13			0.663				0.503
Q14			0.655				0.328
Q15			0.449				0.677
Q16				0.770			0.477
Q17				0.667			0.567
Q18				0.566			0.509
Q19				0.562			0.405
Q20				0.523			0.376
Q21					0.751		0.348
Q22					0.738		0.284
Q23					0.728		0.455
Q24					0.688		0.454
Q25						0.807	0.332
Q26						0.675	0.456
Q27						0.665	0.520

**TABLE 5:** Exploratory Factor Analysis: Factor Statistics and Variance Explained by the Six-Factor Model

Factor	Load	Variance %	Cumulative %	Eigenvalues
1	3.35	12.42	12.4	6.6862
2	2.78	10.30	22.7	2.3586
3	2.72	10.08	32.8	1.7234
4	2.43	8.99	41.8	1.1132
5	2.36	8.73	50.5	0.9903
6	2.04	7.54	58.0	0.5506

**FIGURE 2:**
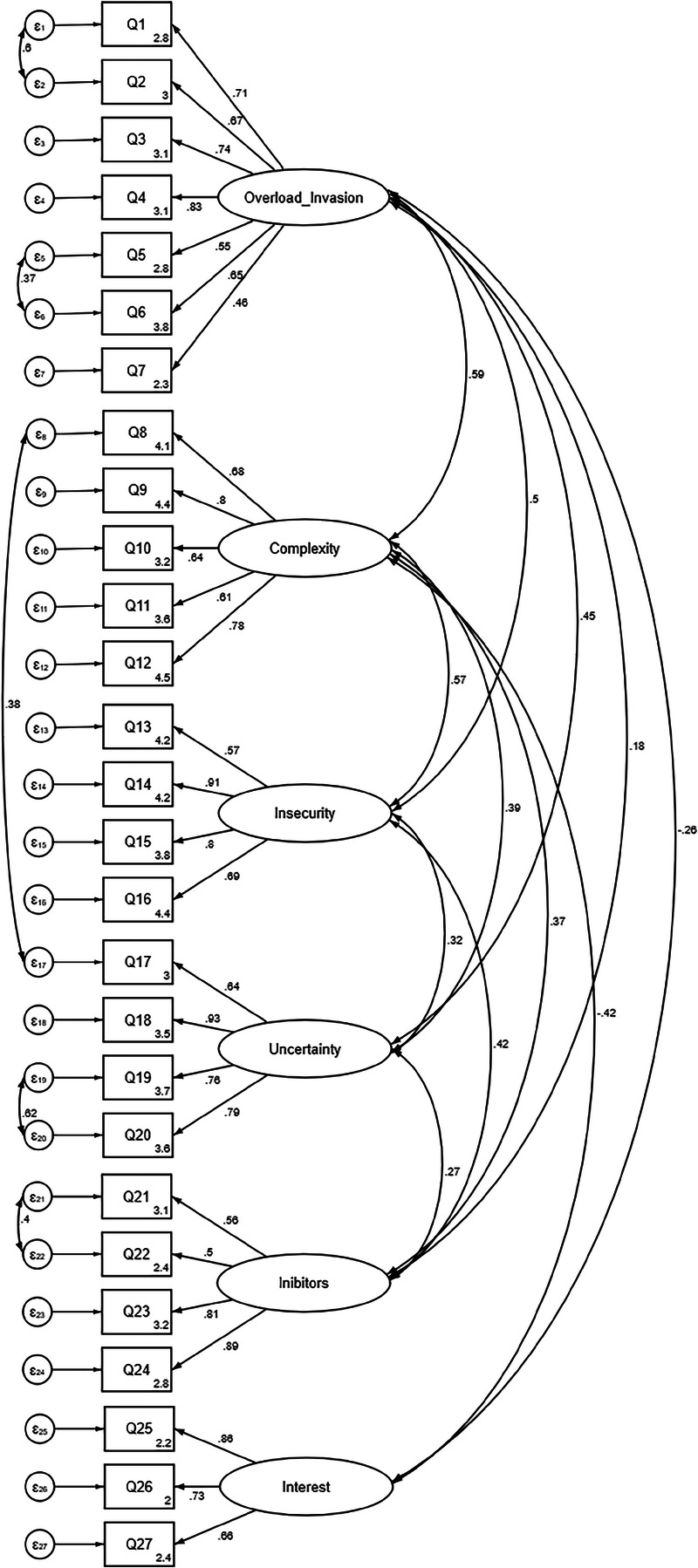
Final CFA model. The coefficients are standardized.

The model was further tested using CFA. The CFA showed good fit indices: *χ*^2^ /*df* = 4.02, comparative fit index = 0.926, Tucker-Lewis index = 0.915, RMSEA = 0.057, and standardized root mean square residual = 0.079. The final CFA model is presented in Figure 2, with standardized coefficients.

## DISCUSSION

Technostress has been reported as an increasing risk in Italian workers, affecting 28% of workers nationally.^21,22,26^ This study aimed to validate the Italian version of the TCS, assessing the reliability of the questionnaire by performing C*α* test and factorial analysis. The back-translated scale has shown good reliability and was validated in Italian.

The EFA highlighted that the first two factors from the original scale (techno-overload and technoinvasion) converged in a single factor.^18^ This could be explained as both of these factors assess how technology affects the personal life of the worker, evaluating the strain that technology has and how it affects the employee’s life out of the work context.^18,44^ Furthermore, the CFA model showed an overall good adaptation of the model.

However, the TCS was translated and validated in many other languages and countries, and the original factors were maintained in the validations performed in China^45^ and in South American countries (Brazil, Peru, Chile).^46^ Therefore, further studies may be needed to investigate if this factorial division is due to the Italian setting or if the techno-overload and technoinvasion factors could be merged to strengthen and shorten the scale. It is noteworthy that a few studies translated and adapted shorter versions of the TCS, extracting only a few items that were useful to their studies.^47,48^ A shorter version of the TCS, still comprehensive of the original factors, could be useful to maintain a well-rounded assessment of the technostress phenomenon, while enhancing participation and response rates (showcased to be higher in shorter surveys^49^).

Furthermore, the TCS could provide an interesting assessment of the mental well-being of workers. As shown in many recent studies, the inability to interact with ICTs in a healthy manner could occur in different situations caused such as information overload (techno overload), intrusion of technology into personal life (techno invasion), or errors and crashes in ICT applications (techno complexity), emerging as an additional factor contributing to work-related stress, potentially compromising the mental well-being of employees.^50^

The Italian validation of the back-translated original scale could represent a starting point to measure the technostress impact within an efficient tool in occupational health surveillance routine in Italy, preventing work-related stress. This instrument could be used by occupational physicians as a routinary screening tool to assess technostress in workers. As the Italian legislation deems the assessment of work-related stress as mandatory,^20^ and considering the emerging issue of technology-related stress in the workplace due to digitalization,^51^ a tool to evaluate technostress is necessary. This validation is a starting point to develop new strategies to assess, evaluate, and prevent technostress in the workplace.

This study has some limitations. One of the limitations is that participation in the study was on a voluntary basis, and the questionnaire itself was distributed via online link and filled out online; therefore, those who did not have the knowledge or means to complete the questionnaire online did not have access to the study. This may have created a selection bias, selecting those participants with higher technological knowledge. Furthermore, the mean age of this study’s participants was relatively young; because technostress has been reported to be higher in older people, this could have created a bias.

In addition, the sample was small, consisting of 90 workers, to perform a validation analysis before the questionnaire is distributed to a larger sample. However, our sample represents a significant segment of the Italian workforce (ample age range, different professions, etc.) rendering our instrument potentially applicable to extensive surveys. Nevertheless, it is prudent to acknowledge that the homogeneity of our sample may limit the generalizability of our findings to other populations. Future research endeavors will be necessary to explore these aspects more comprehensively.

Some methodological limitations should also be acknowledged. First, the relatively small sample size may have affected the stability and reliability of both the exploratory and confirmatory factor analyses. Although there is ongoing debate about minimum sample sizes for factor analysis, many researchers recommend at least 200 to 300 participants for stable factor solutions^52^ or a subject-to-item ratio of 10:1.^53^ Our smaller sample size might have limited the generalizability of the factor structure and potentially affected model fit indices.

Additionally, conducting both EFA and CFA on the same sample, rather than using independent samples for each analysis, may have capitalized on chance characteristics of this specific dataset. A more robust approach would involve using separate samples for the exploratory and confirmatory phases of the analysis.

The limited sample size also restricted our ability to conduct additional validation analyses, such as measurement invariance testing across different subgroups. Future studies should aim to replicate these findings with larger, more diverse samples to confirm the stability of the factor structure identified in this initial validation.

## CONCLUSIONS

The Italian version of the TCS has shown good reliability and was validated in Italian through factor analysis. The CFA model showed a good adaptation of the model. This could be a useful tool in assessing ICT-related stress in Italian workers.

Future studies should include a larger sample of workers to assess the scale of the phenomenon in Italian workers and evaluate the scores related to technostress in this population, to estimate the impact of this issue.

This validation is a starting point to develop new strategies to assess, evaluate, and prevent technostress in the workplace.
